# The Effectiveness of a Sofosbuvir/Daclatasvir Combination in the Treatment of HCV Infection in Patients from Mauritania Undergoing Chronic Hemodialysis

**DOI:** 10.3390/medicina61101753

**Published:** 2025-09-26

**Authors:** Sidi Mohamed Mah, Soufiane Sid’Ahmed, Mohamed Lemrabott, Delahi Welli, Jemal Awa, Abdellatif Sidi Aly, Lionel Rostaing

**Affiliations:** 1 Department of Nephrology, Hemodialysis and Renal Transplantation, National Hospital Center of Nouakchott, Nouakchott BP 612, Mauritania; sidimohamed18@hotmail.com (S.M.M.); medlemrabott09@gmail.com (M.L.); awajemal@gmail.com (J.A.); 2 Faculty of Medicine, Dentistry, and Pharmacy, University of Nouakchott, Nouakchott BP 803, Mauritania; ouldsouf@yahoo.fr (S.S.); abdellatif52@hotmail.com (A.S.A.); 3 Department of Infectious Diseases, National Hospital Center of Nouakchott, Nouakchott BP 612, Mauritania; 4 Department of Gastroenterology and Hepatology, National Hospital Center of Nouakchott, Nouakchott BP 612, Mauritania; dellahi.welli@yahoo.fr; 5 National Council for Organ Donation and Transplantation, Nouakchott BP 557, Mauritania; 6 Department of Nephrology, Dialysis, Apheresis, and Kidney Transplantation, CHU Grenoble-Alpes, 38043 Grenoble, France

**Keywords:** Mauritania, HCV infection, hemodialysis, eradication, sofosbuvir, daclatasvir

## Abstract

*Background and Objectives*: Hepatitis C virus (HCV) infection is highly prevalent among patients undergoing chronic hemodialysis in emerging countries and is associated with significant morbidity and mortality in this population. The objective of this study was to eradicate chronic HCV infection in patients undergoing chronic hemodialysis in Mauritania using a combination of sofosbuvir, 400 mg, and daclatasvir, 60 mg (direct acting antiviral—DAA—therapy), for 3 months. This was a prospective, single-arm, multicenter, interventional study. *Materials and Methods*: A total of 553 patients undergoing hemodialysis were screened for HCV across all hemodialysis centers nationwide. Biological parameters were compared before and after DAA therapy. *Results*: The prevalence of HCV infection was 6.8% (*n* = 38); two patients had undetectable HCV RNA. Out of the 36 eligible patients, 33 received DAA treatment. The median age of the patients was 49 (25–78) years. The average duration on hemodialysis was 9.4 (4–17) years. The median viral load before treatment was 538277 (10–4258571) IU/L. The median alanine aminotransferase (ALT) level was 52.3 (14–278) IU/L. The median hemoglobin level was 10 (6–13) g/dL. HCV viral load was undetectable at week 12 (W12) and week 24 (W24). The sustained virologic response (SVR) rate was 100%. Adverse events included one case of acute pancreatitis, six cases of fatigue (17%), five cases of headache (15%), four cases of diarrhea (12%), three cases of nausea (9%), and four cases of insomnia (12%). *Conclusions*: The combination of sofosbuvir and daclatasvir is effective in patients with HCV undergoing chronic hemodialysis, achieving a 100% SVR rate.

## 1. Introduction

Hepatitis C virus (HCV) infection is highly prevalent among patients undergoing chronic hemodialysis, primarily due to nosocomial transmission [1]. It is associated with significant morbidity and mortality, particularly from cirrhosis and hepatocellular carcinoma [2]. The treatment landscape for HCV has been revolutionized by the advent of direct acting antivirals (DAAs) [3]. However, managing HCV infection in patients with advanced renal failure remains challenging [3]. In developing countries, such as Mauritania, poor hygiene when administering hemodialysis contributes to the high prevalence of chronic HCV infection, e.g., 20% in Africa [4] and 29% in the Middle East and North Africa [5]; this might result in hepatic-related disorders developing into liver cirrhosis. The availably of DAAs worldwide has revolutionized the outcome of chronic HCV infection. However, due to various constraints, the implementation of DAA therapy has been delayed in some countries.

Mauritania is located in West Africa, between the Maghreb and sub-Saharan Africa. Its population is estimated to be 5,315,065 inhabitants. The number of patients on chronic hemodialysis is estimated to be 1200, distributed across 19 dialysis centers. The prevalence of chronic HCV (RNA positive) in the general population of Mauritania was estimated to be 0.75% in 2022 (https://www.globalhep.org/data-profiles/countries/mauritania, access on 22 September 2025).

Among the available DAAs, only the combination of sofosbuvir and daclatasvir is currently accessible in Mauritania. The use of sofosbuvir is generally not recommended in patients with an estimated glomerular filtration rate (eGFR) below 30 mL/min due to the metabolite accumulation resulting from impaired renal clearance [6]. In our study, as demonstrated by studies in HCV-positive patients undergoing hemodialysis [7,8] we used sofosbuvir, 400 mg, after each hemodialysis session and daclatasvir, 60 mg, daily to treat all HCV-positive patients with a detectable viral load.

Our aim was to determine the prevalence of HCV infection with or without HCV replication among patients undergoing chronic hemodialysis in Mauritania and to achieve viral eradication in this population.

## 2. Materials and Methods

This was a prospective, multicenter, interventional study. We included all patients undergoing chronic hemodialysis (HD) three times per week for more than six months. Typically, in every hemodialysis center, there are 2 to 4 shifts per day. All medical hemodialysis devices are single-use and disposable. Regarding hemodialysis machines, they are dedicated either to HCV (+) patients, to HBV (+) patients, or to HCV (−)/HBV (−) patients. The surfaces of the dialysis machines are disinfected between each patient.

When a new patient is referred for hemodialysis in a dialysis facility, HBV and HCV serologies are performed. Thereafter, these serologies are not repeated unless the patient demonstrates an increase in liver enzymes, with these serologies being performed routinely every 3 months in such cases. In our study, patients with positive anti-HCV antibodies underwent quantitative HCV RNA testing by PCR. All patients with a detectable viral load received treatment with sofosbuvir and daclatasvir. Informed consent was obtained from all the included patients. The pre-treatment workup included screening for HBV and HIV coinfections; complete blood count (CBC); liver transaminases; total and conjugated bilirubin; alkaline phosphatase; gamma-glutamyl transferase (GGT); prothrombin time (PT); hepatic ultrasound; electrocardiogram (ECG); and echocardiography. Liver function tests, viral load, and hemoglobin levels were monitored monthly for three months.

Sofosbuvir was administered at a dose of 400 mg after each HD session, i.e., three times a week, and daclatasvir was administered at a dose of 60 mg daily. Sofosbuvir dosing was based on a pharmacokinetic study by Desnoyer et al. [7]. The treatment was scheduled for 3 months, including those with liver cirrhosis. HCV RNA viral load was measured at baseline, at the end of treatment, and three months post-treatment.

In addition, all the patients undergoing hemodialysis were tested for hepatitis B virus (HBV); only those in whom HBV DNA was positive were placed under anti-HBV therapy.

Because the liver FibroScan was self-paid by the patients, the diagnosis of liver cirrhosis was based on clinical grounds such as splenomegaly, esophageal varices, thrombopenia, and portal hypertension on abdominal echo doppler.

Patients were followed up one week after completing treatment, and then monthly for three months, for clinical and biological monitoring of adverse events.

Ethical review and approval were waived because eradication of HCV infection around the world has been implemented by the World Health Organization (https://www.who.int/health-topics/hepatitis/elimination-of-hepatitis-by-2030#tab=tab_1, access on 22 September 2025).

### Statistical Analyses

Patient medical records and dialysis charts were sources of data collection. The data collection tools included socio-demographic characteristics (such as age and sex), patient clinical characteristics, e.g., features of liver cirrhosis, and laboratory features (such as hemoglobin, alanine aminotransferase (ALT), and aspartate aminotransferase (AST)). Categorical variables were presented as frequencies and percentages, while continuous variables were described using the median and ranges. Categorical data were compared using the chi-squared test.

## 3. Results

A total of 553 patients were screened for HCV across all hemodialysis (HD) centers in the country. Thirty-eight (38) patients tested positive for HCV serology, corresponding to a prevalence of 6.8%; out of these, two had undetectable HCV RNA. Two patients had HBV coinfection. HBV DNA was negative; therefore, they did not undergo anti-HBV therapy. In addition, both were in the cirrhotic stage. No patients were infected with HIV. Of the 36 eligible patients, only 33 received treatment, as 2 died before treatment initiation and 1 refused treatment. Table 1 displays the baseline characteristics of the population. The mean age of the patients was 49 years. The mean duration on HD was 114 months. Initial nephropathy was vascular in 13 cases (34%), diabetic nephropathy in 8 cases (21%), chronic glomerulonephritis in 6 cases (15%), polycystic kidney disease in 2 cases (5%), and 11 patients (28%) had undetermined nephropathy.

The mean HCV viral load was 538,277 IU/L (range: 10–4,258,571 IU/L). The mean ALT level prior to treatment was 52.3 IU/L (range: 14–278 IU/L). The mean hemoglobin level before treatment was 10 g/dL (range: 9–13 g/dL).

The treatment duration was three months (12 weeks). All the treated patients completed their treatment. As compared to baseline values, at the end of DAA therapy, there were no significant changes regarding AST [55 (35–138) vs. 48 (15–120) IU/L], and ALT [60 (48–286) vs. 52.3 (14–278) IU/L)] values. Conversely, HCV RNA was undetectable at week 12 including in the two HBV-coinfected patients and remained undetectable at week 24. Sustained virological response (SVR), defined as an undetectable viral load 12 weeks after the end of treatment, was achieved in 100% of cases.

Regarding adverse events, there was no significant change for hemoglobin levels, i.e., 105 (60–140) g/L at the end of DAA therapy vs. 100 (60–130) g/L at baseline. Only one patient experienced an episode of acute pancreatitis, with abdominal pain and elevated serum amylase levels, leading to treatment discontinuation. The clinical outcome was favorable. Table 2 presents the main adverse effects.

## 4. Discussion

This study represents the first experience with the use of direct acting antivirals (DAAs) in HCV-positive patients undergoing chronic hemodialysis (HD) in Mauritania. The DAAs recommended for patients on dialysis include elbasvir/grazoprevir [9,10] and glecaprevir/pibrentasvir [11,12]; however, these agents are not available in Mauritania. The only DAAs currently accessible are sofosbuvir and daclatasvir. Sofosbuvir use is not generally recommended in patients undergoing hemodialysis due to metabolite accumulation [4]. Recently, Chariyavilaskul et al. published a study on sofosbuvir/velpatasvir (SOF/VEL) in 12 Thai HCV/RNA-positive patients undergoing hemodialysis with an emphasis on the pharmacokinetics of SOF/VEL and its metabolites, GS-331007 [13]. There were no differences in the SOF/VEL pharmacokinetic parameters between on- and off- dialysis days. On the contrary, GS-331007 exhibited a 30% reduction in the area under the plasma concentration–time curve from time 0 to 24 h (AUC0–24 h) on dialysis days compared to non-dialysis days (AUC0–24 h ratio 0.68 vs. 1.04, respectively). In addition, the dialysis clearance of SOF and GS-331007 was 9.35 (8.72–15.11) and 8.89 (8.52–14.07) mL/min, respectively. Based on these results, the authors subsequently administered an alternate-day regimen of SOF/VEL (400/100 mg) to their patients for 12 weeks. This resulted in an undetectable plasma HCV viral load without side effects.

Shehadeh et al. performed a systematic review and meta-analysis on the efficacy and safety of sofosbuvir in the treatment of hepatitis C among patients undergoing hemodialysis [14]. They found that the overall pooled estimate of the efficacy of SOF-based therapy was 95% (95% CI 91–98%); in addition, the efficacy of the SOF-based regimen was 92% (95% CI 80–99%), 98% (95% CI 96–100%), and 100% (95% CI 95–100%) for the following doses: 400 mg on alternate days, 400 mg daily, and 200 mg daily, respectively. Regarding the side effects, the most frequent was fatigue, with a pooled prevalence of 16% (95% CI 5–29%), followed by anemia, 15% (95% CI 3–31%), and nausea or vomiting, 14% (95% CI 4–27%). The authors concluded that SOF-based regimens in the treatment of HCV infection among patients undergoing hemodialysis are both effective and safe. Kawakami et al. studied daclatasvir pharmacokinetics in patients with HCV undergoing hemodialysis. They found that the area under the plasma concentration–time curve of daclatasvir from 0 to 6 h (AUC0–6 h) was slightly lower in patients undergoing hemodialysis than in patients with normal renal function; however, the difference was not statistically significant [15].

In our study, all patients received sofosbuvir, 400 mg, after each hemodialysis session and daclatasvir, 60 mg, daily for 12 weeks. Only one serious adverse event, an episode of acute pancreatitis, was observed, with a favorable clinical outcome after treatment discontinuation. All other adverse effects were minor. Similarly, Kc et al. reported 3 cases of gall stone pancreatitis in 181 HCV/RNA-positive patients with normal renal function who were treated with sofosbuvir-based DAA with or without daclatasvir [16]. With the combination of sofosbuvir plus daclatasvir, we achieved a 100% sustained virological response (SVR), resulting in the eradication of hepatitis C across all dialysis centers in our country.

Similar studies have reported the comparable efficacy and good tolerability of this regimen in patients undergoing chronic HD [5,17]. However, a recent case series from Pakistan showed that among 21 HCV-positive patients undergoing hemodialysis who were treated with sofosbuvir-based therapy, only 91.3% achieved viral eradication on completion of treatment, and 2 were non-responders. In addition, two relapsed after the end of therapy [18]. The limitations of our study include the absence of HCV genotyping, the lack of measurement of sofosbuvir metabolites, and the relatively small sample size. Nevertheless, this study represents a significant contribution toward the eradication of hepatitis C virus infection in hemodialysis centers.

Indeed, in hemodialysis units from developing countries, the causes of HCV infection were mostly nosocomial transmission within the hemodialysis facility and blood transfusions [19,20,21]. Recently, Huang et al. from Taïwan reported a prospective HCV elimination program for patients undergoing hemodialysis. There were two surveys across 22 hemodialysis centers (with more than 2300 patients): one in 2019 with the implementation of DAA therapy for HCV-positive patients undergoing hemodialysis and one in 2021 [22]. In 2019, 7.7% of patients undergoing hemodialysis were HCV-viremic; of 152 patients successfully linked to treatment with DAA, 140 (92.1%) patients achieved a sustained virological response. In 2021, 1733 patients participated in a second round of surveillance; five anti-HCV-negative patients experienced anti-HCV seroconversion. Of 119 DAA-cured patients and 102 spontaneous HCV clearance patients, none had HCV reinfection. Therefore, the annual incidence of new HCV infection was 0.1%. Eventually, the overall HCV-viremic rate decreased from 7.7% in 2019 to 0.6% in 2021. At the institutional level, 45.5% (10/22) eradicated HCV, and 82% (18/22) of HD units had no new HCV infections or reinfections. The conclusion is that DAA therapy has a preventative role toward HCV elimination via maintenance hemodialysis. However, this might be different in other settings. Recently, Sarhan et al. reported on the prevalence of HCV infection in hemodialysis units in Egypt [23]. Indeed, the prevalence of chronic HCV infection is very high in the Egyptian population; in 2016, the Egyptian government implemented a large and ambitious DAA therapy program in order to eradicate HCV infection by 2030 [24]. In a multicenter, cross-sectional study conducted during the period from November 2022 to April 2023, Sahran et al. included 500 patients undergoing hemodialysis, finding that 23.4% of patients were previously infected with HCV. Of these, 12.6% received treatment, and 10.8% did not receive treatment due to a variety of reasons. The authors conclude that the low prevalence of HCV among patients undergoing hemodialysis confirms that HCV infection is not currently a significant health concern among patients on maintenance hemodialysis [24].

The strength of our study is that all patients from Mauritania undergoing chronic hemodialysis were screened for HCV infection; had they had ongoing HCV replication, they were accordingly treated with DAAs under the initiative to eradicate HCV in hemodialysis facilities from the country. The limitations of this study are (i) the lack of a new HCV survey across Mauritanian hemodialysis facilities and (ii) the lack of novelty regarding the efficacy of DAA therapy in the population undergoing dialysis.

## 5. Conclusions

The combination of sofosbuvir and daclatasvir when implemented universally in hemodialysis facilities in a low-income country such as Mauritania is effective in treating chronic hepatitis C virus infection in patients undergoing hemodialysis. In addition, it allows for the eradication of HCV infection in patients undergoing hemodialysis because no longer is there nosocomial transmission.

## Figures and Tables

**Table 1:** Baseline characteristics of the HCV-positive patients undergoing hemodialysis.

Number of Patients	38
Age (years)	49 (25–78)
Gender (M/F)	19/19
Duration on HD (years)	9.4 (4–17)
AVF (n)	37
Permanent catheter	1
History of blood transfusion (yes)	32
Diabetic nephropathy (n)	8
HIV (n)	0
HBV (n)	2
Hemoglobin (g/dL)	10 (6–13)
Albumin (g/L)	38.7 (19.2–48.4)
AST (IU/L)	48 (15–120)
ALT (IU/L)	52.3 (14–278)
HCV RNA viral load (IU/L)	538,277 (10–4,258,571)

Abbreviations: HCV: hepatitis C virus; HIV: human immunodeficiency virus; HBV: hepatitis B virus; AVF: arteriovenous fistula; M: male; F: female; AST: aspartate aminotransferase; ALT: alanine aminotransferase.

**Table 2:** Adverse events during DAA therapy.

Adverse Event	Number of DAA-Treated Patients	Percentage
Acute pancreatitis	1	3%
Fatigue	6	17%
Insomnia	4	12%
Diarrhea	5	15%
Headaches	3	9%
Nausea	4	12%

Abbreviations: DAA, direct acting antiviral.

## Data Availability

Data are available upon reasonable request.

## References

[1] Jadoul M., Bieber B.A., Martin P., Akiba T., Nwankwo C., Arduino J.M., Goodkin D.A., Pisoni R.L. (2019). Prevalence, incidence, and risk factors for hepatitis C virus infection in hemodialysis patients. Kidney Int..

[2] Kenfack-Momo R., Ngounoue M.D., Kenmoe S., Takuissu G.R., Ebogo-Belobo J.T., Kengne-Ndé C., Mbaga D.S., Menkem E.Z., Fogang R.L., Tchatchouang S. (2024). Global epidemiology of hepatitis C virus in dialysis patients: A systematic review and meta-analysis. PLoS ONE.

[3] Poustchi H., Majd Jabbari S., Merat S., Sharifi A., Shayesteh A.A., Shayesteh E., Minakari M., Fattahi M.R., Moini M., Roozbeh F. (2020). The combination of sofosbuvir and daclatasvir is effective and safe in treating patients with hepatitis C and severe renal impairment. J. Gastroenterol. Hepatol..

[4] Harfouche M., Chemaitelly H., Mahmud S., Chaabna K., Kouyoumjian S.P., Al Kanaani Z., Abu-Raddad L.J. (2017). Epidemiology of hepatitis C virus among hemodialysis patients in the Middle East and North Africa: Systematic syntheses, meta-analyses, and meta-regressions. Epidemiol. Infect..

[5] Adane T., Getawa S. (2021). The prevalence and associated factors of hepatitis B and C virus in hemodialysis patients in Africa: A systematic review and meta-analysis. PLoS ONE.

[6] AASLD-IDSA HCV Guidance Panel (2018). Hepatitis C Hepatitis C Guidance 2018 Update: AASLD-IDSA recommendations for testing, managing, and treating hepatitis C virus infection. Clin. Infect. Dis..

[7] Desnoyer A., Pospai D., Lê M.P., Gervais A., Heurgué-Berlot A., Laradi A., Harent S., Pinto A., Salmon D., Hillaire S. (2016). Pharmacokinetics, safety and efficacy of a full dose sofosbuvir-based regimen given daily in hemodialysis patients with chronic hepatitis C. J. Hepatol..

[8] Llenas-García J., Padilla S., Masiá M., Gutiérrez F. (2018). Sofosbuvir plus daclatasvir as an alternative for patients on haemodialysis with genotype 2 hepatitis C virus infection. Enfermedades Infecc. Microbiol. Clin. Engl. Ed..

[9] Liu C.H., Peng C.Y., Fang Y.J., Kao W.-Y., Yang S.-S., Lin C.-K., Lai H.-C., Su W.-P., Fang S.-U., Chang C.-C. (2020). Elbasvir/grazoprevir for hepatitis C virus genotype 1b East-Asian patients receiving hemodialysis. Sci. Rep..

[10] Ji Q., Chu X., Zhou Y., Liu X., Zhao W., Ye W. (2022). Safery and efficacy of grazoprevir/elbasvir in the treatment of acute hepatitis C in hemodialysis patients. J. Med. Virol..

[11] Morishita A., Ogawa C., Moriya A., Tani J., Yoneyama H., Fujita K., Oryu M., Senoo T., Takaguchi K., Masaki T. (2020). Clinical outcomes of hepatitis C virus elimination using glecaprevir and pibrentasvir in hemodialysis patients: A multicenter study. Hepatol. Res..

[12] Miyasaka A., Yoshida Y., Murakami A., Hoshino T., Sawara K., Numao H., Takikawa Y. (2022). Safety and efficacy of glecaprevir and pibrentasvir in north Tohoku Japanese patients with genotype ½ hepatitis C virus infection. Health Sci. Rep..

[13] Chariyavilaskul P., Prompila N., Wittayalertpanya S., Lekhyananda S., Prasithsirikul W., Trakarnvanich T., Jeenapongsa S., Susantitaphong P., Kerr S., Avihingsanon A. (2024). Pharmacokinetics of Sofosbuvir/Velpatasvir and efficacy of an alternate-day treatment in hemodialysis patients with chronic hepatitis C infection. Clin. Transl. Sci..

[14] Shehadeh F., Kalligeros M., Byrd K., Shemin D., Mylonakis E., Martin P., D’aGata E.M.C. (2020). Efficacy and safety of sofosbuvir in the treatment of hep C among patients on hemodialysis: A systematic review and meta-analysis. Sci. Rep..

[15] Kawakami Y., Imamura M., Ikeda H., Suzuki M., Arataki K., Moriishi M., Mori N., Kokoroishi K., Katamura Y., Ezaki T. (2016). Pharmacokinetics, efficacy and safety of daclatasvir plus asunaprevir in dialysis patients with chronic hepatitis C: Pilot study. J. Viral Hepat..

[16] Kc S., Karki N., Sharma D., Khadka S., Tiwari P.S. (2021). Direct-Acting Antivirals in the Treatment of Hepatitis C Virus (HCV)/Human Immunodeficiency Virus (HIV) Co-infected Patients: Real-Life Experience From Nepal. Cureus.

[17] Tronina O., Durlik M., Orłowska I., Lorenc B., Łapiński T.W., Garlicki A., Dybowska D., Zarębska-Michaluk D., Tudrujek-Zdunek M., Citko J. (2021). Real-world direct-acting antiviral treatment in kidney transplant and hemodialysis patients: The EpiTer-2 multicenter observational study. Ann Gastroenterol..

[18] Salim A., Farooq M.O., Mengal F.U.A., Malik K. (2020). Sofosbuvir-based Treatment for HCV: A Safe Option in Patients Undergoing Hemodialysis. J. Coll. Physicians Surg. Pak..

[19] Izopet J., Pasquier C., Sandres K., Puel J., Rostaing L. (1999). Molecular evidence for nosocomial transmission of hepatitis C virus in a French hemodialysis unit. J. Med. Virol..

[20] Hmaïed F., Ben Mamou M., Dubois M., Pasquier C., Sandres-Saune K., Rostaing L., Slim A., Arrouji Z., Ben Redjeb S., Izopet J. (2007). Determining the source of nosocomial transmission in hemodialysis units in Tunisia by sequencing NS5B and E2 sequences of HCV. J. Med. Virol..

[21] Amjad U., Ahmad S.Q., Mir S., Ayub M. (2020). Association of anti-HCV sero-prevalence with blood transfusion and practice of haemodialysis from multiple centres in patients on maintenance haemodialysis. Pak. J. Med. Sci..

[22] Huang C.F., Dai C.Y., Wang C.W., Liang P.-C., Wei Y.-J., Tsai P.-C., Jang T.-Y., Hsu P.-Y., Lee J.-J., Niu S.-W. (2023). FORMOSA-LIKE investigators. Therapy as prevention toward HCV elimination in maintenance hemodialysis: A multi-center, prospective cohort study. Clin. Kidney J..

[23] Sarhan I.I., El Sharkawy M.M., El Gaafary M.M., Hendam D.M.T., Gouda K. (2024). The burden of HCV among prevalent hemodialysis patients after the National Egyptian HCV Eradication program. Egypt. J. Immunol..

[24] Gomaa A., Gomaa M., Allam N., Waked I. (2024). Hepatitis C elimination in Egypt: Story of success. Pathogens.

